# Reversible LSD1 inhibition with HCI-2509 induces the p53 gene expression signature and disrupts the MYCN signature in high-risk neuroblastoma cells

**DOI:** 10.18632/oncotarget.24035

**Published:** 2018-01-06

**Authors:** Sumati Gupta, Kelly Doyle, Timothy L. Mosbruger, Andrew Butterfield, Alexis Weston, Allison Ast, Mohan Kaadige, Anupam Verma, Sunil Sharma

**Affiliations:** ^1^ Huntsman Cancer Institute, University of Utah, Salt Lake City, Utah, USA; ^2^ Central Laboratory, Intermountain Healthcare, Murray, Utah, USA; ^3^ Department of Pathology, Primary Children’s Hospital, Salt Lake City, Utah, USA; ^4^ Department of Pediatric Hematology/Oncology, Primary Children’s Hospital, Salt Lake City, Utah, USA

**Keywords:** neuroblastoma, MYCN, LSD1, HCI-2509, p53

## Abstract

Lysine-Specific Demethylase 1 (LSD1) over-expression correlates with poorly differentiated neuroblastoma and predicts poor outcome despite multimodal therapy. We have studied the efficacy of reversible and specific LSD1 inhibition with HCI-2509 in neuroblastoma cell lines and particularly the effect of HCI-2509 on the transcriptomic profile in MYCN amplified NGP cells. Cell survival assays show that HCI-2509 is cytotoxic to poorly differentiated neuroblastoma cell lines in low micromole or lower doses. Transcriptional profiling of NGP cells treated with HCI-2509 shows a significant effect on p53, cell cycle, MYCN and hypoxia pathway gene sets. HCI-2509 results in increased histone methyl marks and p53 levels along with cell cycle arrest in the G2/M phase and inhibition of colony formation of NGP cells. Our findings indicate that LSD1 inhibition with HCI-2509 has a multi-target effect in neuroblastoma cell lines, mediated in part via p53. MYCN-amplified neuroblastoma cells have a targeted benefit as HCI-2509 downregulates the MYCN upregulated gene set.

## INTRODUCTION

Neuroblastoma is the most common extracranial solid tumor in childhood. More than 650 cases of neuroblastoma are diagnosed each year in North America [1]. The clinical course of neuroblastoma ranges from low-grade disease with spontaneous remission to high-grade disease with poor outcome. Treatment modalities include surgery, chemotherapy, radiation, myeloablative therapy followed by autologous hematopoietic stem cell transplant, anti-GD2 antibody, Interleukin-2, GM-CSF and retinoic acid [2]. The 5-year survival rate for children diagnosed with neuroblastoma from 2006-2012 was estimated to be close to 77 percent [3]. The 5-year survival rate for children with neuroblastoma in the high-risk group (risk category per the Children’s Oncology Group criteria) is only 40% to 50% [4].

Neuroblastoma is an embryonal malignancy that originates from undifferentiated neural crest progenitor cells. The clinical course of neuroblastoma ranges from spontaneous regression to treatment refractory disease state, with a relatively non-explanatory genomic landscape, indicating a significant role of epigenetic modulation. Neuroblastoma with poor prognosis have been characterized by a high-risk functional MYCN signature. These have increased expression of MYCN inducible genes by either MYCN amplification or increased nuclear localization of MYCN due to MYCN stabilization. MYCN induces cell cycle progression and represses neuronal differentiation [5]. Trimethylated fourth and twenty-seventh lysine residues on histone protein H3 (H3K4me3, H3K27me3) exert the opposing influences of gene activation and repression on the promoters of oncogenes *MYCN* and *ARID1B*. A switch in the histone code from the repressive H3K27me3 mark to the activated H3K4me3 mark has been noted on the promoters of *MYCN* and *ARID3B* in high risk neuroblastoma [6]. This results in overexpression of *MYCN* and *ARID3B.* Conversely, during the embryologic development of sympathetic ganglia, *MYCN* expression is lost in the neural crest cells due to a histone code switch at *MYCN* and *ARID3B* gene promotor from H3K4me3 to the repressive H3K27me3 [6]. Moreover, MYCN exerts an epigenetic influence in neuroblastoma via several mechanisms involving DNA and histone methylation, and deacetylation [7].

Lysine(K)-specific demethylase 1 (LSD1, also known as KDM1A and AOF2), the first identified histone demethylase, is a flavin-dependent monoamine oxidase [8]. LSD1 selectively demethylates the di- and mono-methylated fourth and ninth lysine residues on histone protein H3 (H3K4me2/me1 and H3K9me2/me1). The substrate specificity of LSD1 with its downstream effect on gene expression depends on its associated co-factors. LSD1 functions as a co-repressor when it partners with CoREST (co-repressor for element-1-silencing transcription factor) [9] and NuRD (nucleosome remodeling and deacetylation) [10] complexes and causes demethylation of H3K4me2 and H3K4me1. In partnership with nuclear receptors, LSD1 functions as an activator of gene expression by demethylation of H3K9me2 and H3K9me1 [11]. LSD1 has a significant role in embryologic development and differentiation. It influences the expression of developmental genes by regulation of H3K4di-/tri-methyl marks. LSD1 directs the histone code to maintain the silencing of several developmental genes in human embryonic stem cells and so maintains the pluripotency of embryonic stem cells (ESCs) [12]. Besides histone modification, LSD1 also demethylates specific lysine residues on several non-histone proteins such as p53, E2F1, MYPT1 and DNMT1 [13], thus affecting cell cycle progression and gene expression.

LSD1 is overexpressed in several malignancies including lung, breast and prostate cancers and correlates with poorly differentiated advanced disease status with reduced survival [14]. Tissue microarray studies of poorly differentiated neuroblastoma have demonstrated a significantly higher degree of LSD1 expression in these tumors. Furthermore, LSD1 mRNA expression in tumors correlates with poorer event-free survival. Interestingly, LSD1 protein expression does not correlate with MYCN amplification [15]. LSD1 protein is overexpressed in poorly differentiated neuroblastoma cell lines. Induction of differentiation with ATRA leads to decrease in LSD1 levels in these cell lines. LSD1 inhibition with siRNA and small molecule inhibitors from the monoamine oxidase inhibitor (MAOI) category (pargyline, tranylcypromine, and clorgyline) causes differentiation and inhibits the growth of neuroblastoma cell lines and xenografts [15]. LSD1 inhibition with siRNA has been shown to cause SH-SY5Y cell death as well as enhance the ability of retinoic acid to differentiate and lead to the death of SH-SY5Y cells [16]. MiR-137 is a microRNA that downregulates expression of LSD1 in neuroblastoma and leads to tumor suppression [17]. E3 ubiquitin ligase, Jade-2, negatively regulates LSD1 and has been proposed as a potential anti-cancer treatment strategy in neuroblastoma [18]. LSD1 is a binding partner of MYCN and influences the expression of tumor suppressor genes repressed by MYCN [19]. LSD1 inhibition has been shown to reduce MYCN-driven NDRG1 regulation, which affects epithelial-mesenchymal transition [20]. Targeting LSD1 in high- risk neuroblastoma remains an ongoing effort.

The benzamide group of potent, specific and reversible small molecule inhibitors of LSD1 were designed and developed to be very specific to LSD1 and have little off-target activity compared to tranylcypromine [21]. HCI-2509, a prototype of this group has an IC50 of 13 nM against LSD1. HCI-2509 has remarkable single agent efficacy and *in vivo* tolerability in other poorly differentiated malignancies - Ewing’s sarcoma [22], endometrial cancer [23], and prostate cancer [24]. In Ewing’s sarcoma specifically, HCI-2509 disrupts the transcriptional activity of EWS/FLI fusion protein [22].

In this study, we evaluate the effect of LSD1 inhibition with HCI-2509 in poorly differentiated neuroblastoma cell lines and examine the global transcriptomic changes induced by HCI-2509 to elucidate the mechanisms of the efficacy of HCI-2509 in MYCN amplified neuroblastoma cells.

## RESULTS

### HCI-2509 inhibits the growth of neuroblastoma cell lines in a dose dependent manner

**Figure 1:**
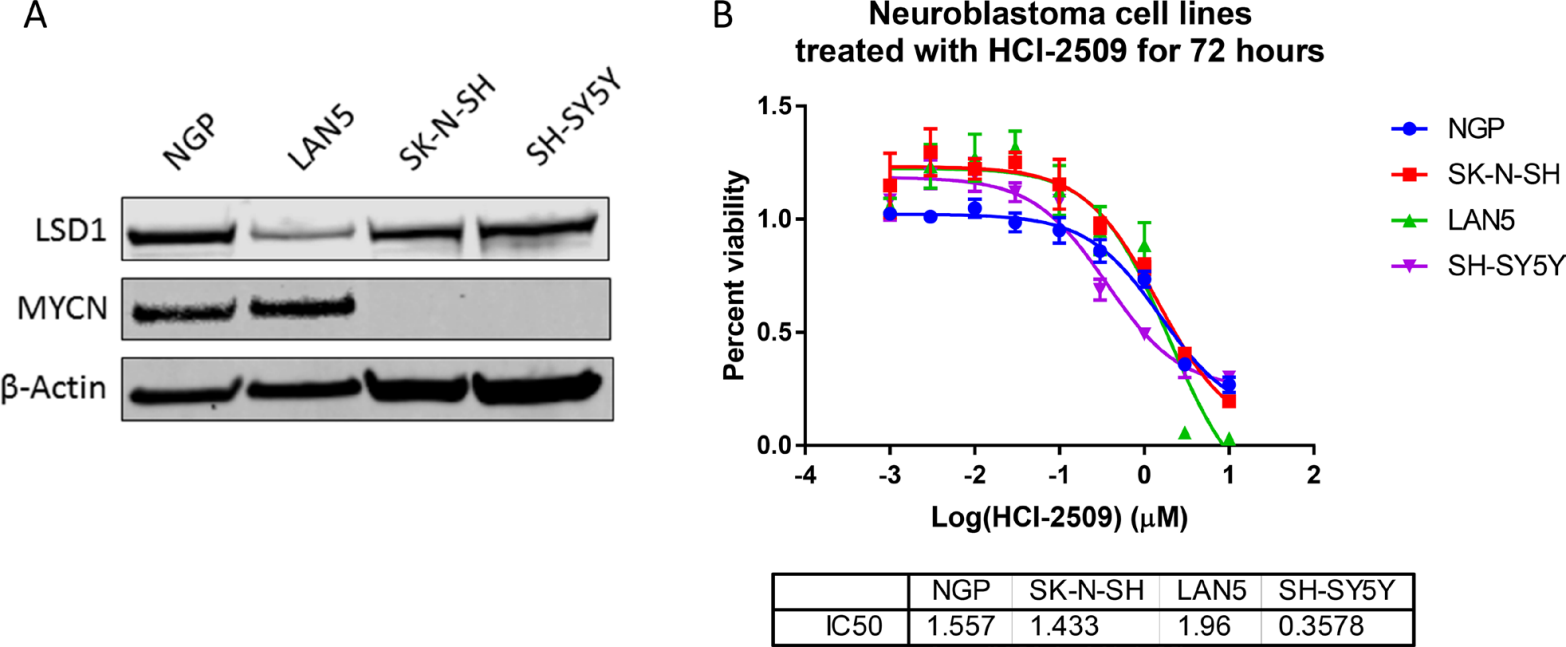
The effect of HCI-2509 on the cell viability of MYCN amplified and not amplified neuroblastoma cell lines after 72 hours of treatment (**A**) Western Blot to show MYCN expression and LSD1 expression in four different neuroblastoma cell lines. NGP and LAN5 cells are MYCN amplified, and SH-SY5Y and SK-N-SH are not MYCN amplified. (**B**) Cell viability assays to assess the growth inhibitory effects of HCI-2509 in MYCN-amplified and non-MYCN-amplified neuroblastoma cell lines. Dose response curves showing percent viability normalized to vehicle controls after 72 hours of treatment plotted against the logarithmic drug concentrations. Half-maximal inhibitory concentrations (IC50s) were calculated using GraphPad Prism. The error bars represent biological replicates.

To evaluate the effect of HCI-2509 on various poorly differentiated neuroblastoma cell lines, we studied cell lines that are MYCN amplified (LAN5 and NGP) and non-MYCN-amplified (SH-SY5Y and SK-N-SH). Consistent with previous reports [25, 26], expression of MYCN was high in LAN5 and NGP cells and expression of LSD1 was observed in all four cell lines (Figure 1A). HCI-2509 was cytotoxic to neuroblastoma cell lines SH-SY5Y, SK-N-SH, LAN5 and NGP with 72 hour IC50s in high nanomole to low micromole concentrations (Figure 1B). These results suggest that LSD1 inhibition is likely to be an effective therapeutic strategy in poorly differentiated neuroblastoma regardless of MYCN amplification status.

### Transcriptomic profiling suggests a sustained effect on p53, cell cycle, metabolic and MYCN gene sets in NGP cells

Because MYCN overexpression has been identified as a strong predictor of poor outcomes in neuroblastoma we investigated the effects of HCI-2509 on NGP cells- a MYCN amplified neuroblastoma cell line. To identify the early and late gene expression changes upon treatment, transcriptomic profiling was performed on cells that were treated for 4 and 24 hours with HCI-2509.

**Figure 2:**
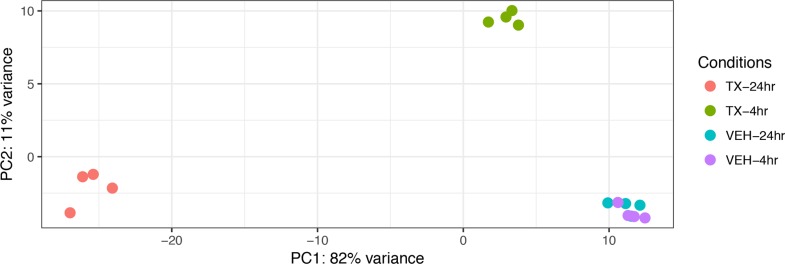
A Principal Components Analysis (PCA) of gene expression data from vehicle treated and drug (3 µM HCI-2509) treated NGP cells at 4 hours and 24 hours to assess early and late gene expression change Each time point and treatment condition had four biological replicates. PC = Principal component. The Euclidean distance between the gene expression profiles of each treatment condition is measured using the variance of stabilized, normalized counts for all genes. The higher the PC number, the more different two samples are from each other. The gene expression of the vehicle treated samples at 4 hours and 24 hours (VEH-4 hr, VEH-24 hr) clusters close together with minimal variance. PC1 contains the most variability, PC2 contains the second most variability. The gene expression of the 24 hour treated samples (TX-24 hr) is driving PC1 and the gene expression of the 4 hour treated samples (TX-4 hr) is driving PC2, which is stronger.

**Figure 3:**
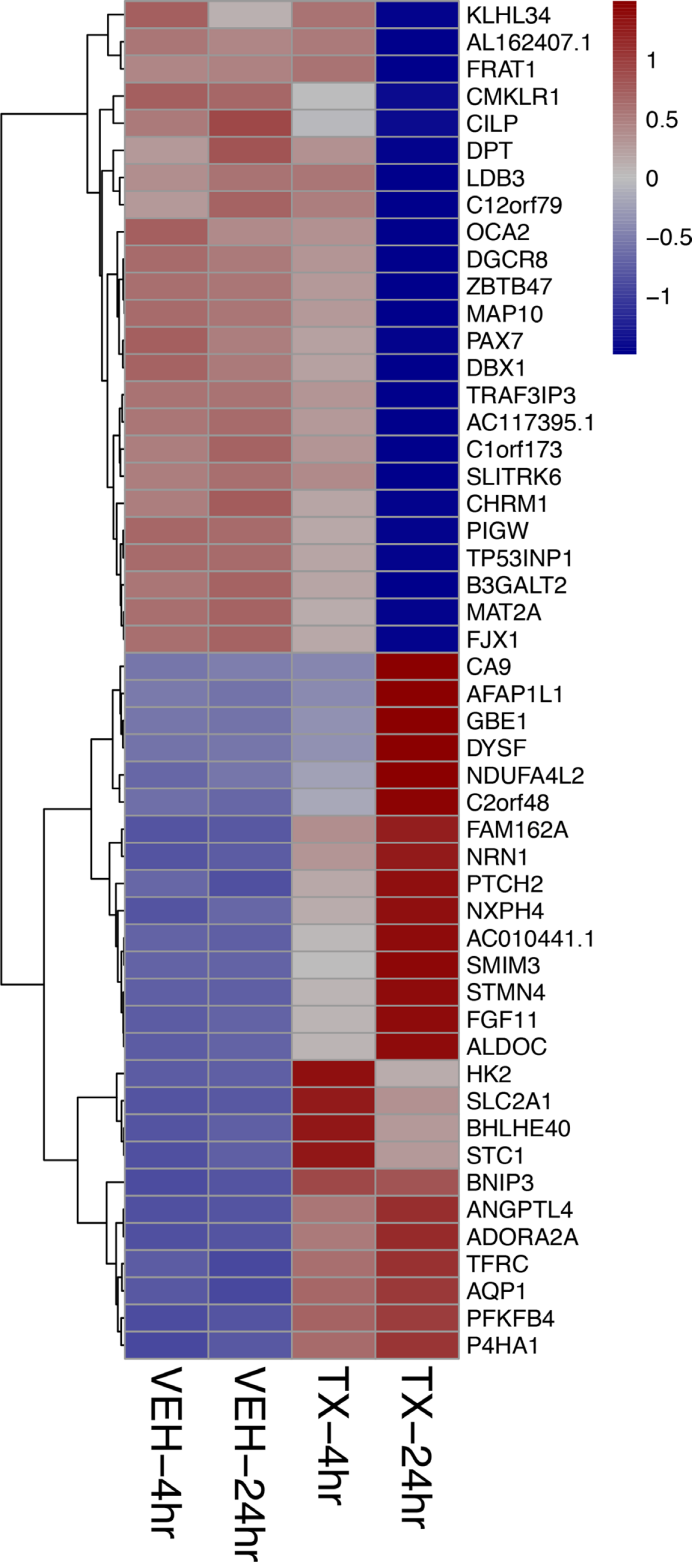
Heat map showing the top 50 of all protein coding genes affected by HCI-2509 at 4 and 24 hours of treatment The strongest log2-fold change of all protein coding genes across either time point (4, 24 hours) post treatment of NGP cells with HCI-2509 as compared to vehicle.TX-24 hr = treatment with 3 µM of HCI-2509 for 24 hours, VEH-4hr = vehicle treatment for 4 hours The genes are ordered by fold change.

A Principal Component Analysis of the Euclidean distance in the gene expression of the four different treatment conditions: 4-hour vehicle, 4-hour treatment, 24-hour vehicle and 24-hour treatment is represented graphically in Figure 2. The biological replicates of each treatment condition cluster close together indicating reproducibility. The vehicle treatment replicates at 4 and 24 hours each cluster together as well. Principal Component 1 (PC1) represents the most variance (82%) and is powered by the 24-hr treatment samples. Principal Component 2 (PC2) contains the second most variance (11%) and is driven by the samples at the 4 hour treatment time point. To analyze the overall effect on various biological processes we created a heat map of the top 50 most affected genes after 4 and 24 hours of treatment with HCI-2509. It is noted that genes that belong to the glycolytic pathway and that lead to neurite formation are among the top 50 upregulated genes (Figure 3).

To assess the evolution of gene expression change and specifically identify the pathways that drive a sustained cytotoxic effect on the NGP cells when treated with HCI-2509, the Hallmark gene sets of the Mutational Signatures Database (MSigDB) were referenced for enrichment. The Hallmark gene sets represent distinct well-defined biological states with minimization of overlap and redundancy.

The MSigDB Hallmark gene sets that were significantly affected (highest ranked, significantly affected signatures in the up and down direction, with an FDR of <25%) were compared at 4 hour and 24 hour time points. The P53 and Hypoxia gene signatures were consistently upregulated at 4 and 24 hours of treatment with HCI-2509, and G2M checkpoint gene signature was downregulated at both the 4 and 24 hour time points with an FDR <25% (Table 1).

**Table 1:** The significantly affected (up and downregulated) Hallmark gene sets enriched in a sustained manner at 4 hours and 24 hours of HCI-2509 treatment (FDR *q* value less than 25%)

GeneSets	Time point	Correlation	Direction	*P* Value	FDR
HALLMARK_G2M_CHECKPOINT	4 hou r	0.01	Down	1.78E-12	4.44E-11
HALLMARK_G2M_CHECKPOINT	24 hour	0.01	Down	0.039872	0.13915
HALLMARK_HYPOXIA	4 hour	0.01	Up	2.56E-09	4.26E-08
HALLMARK_HYPOXIA	24 hour	0.01	Up	7.86E-06	0.000131
HALLMARK_P53_PATHWAY	4 hour	0.01	Up	0.109509	0.245777
HALLMARK_P53_PATHWAY	24 hour	0.01	Up	0.041745	0.13915

In the context of MYCN amplified neuroblastoma, p53 and MYCN play a major role in maintaining the poorly differentiated state and cell proliferation [27], but the MYCN gene signatures are not part of the Hallmark gene sets.

**Figure 4:**
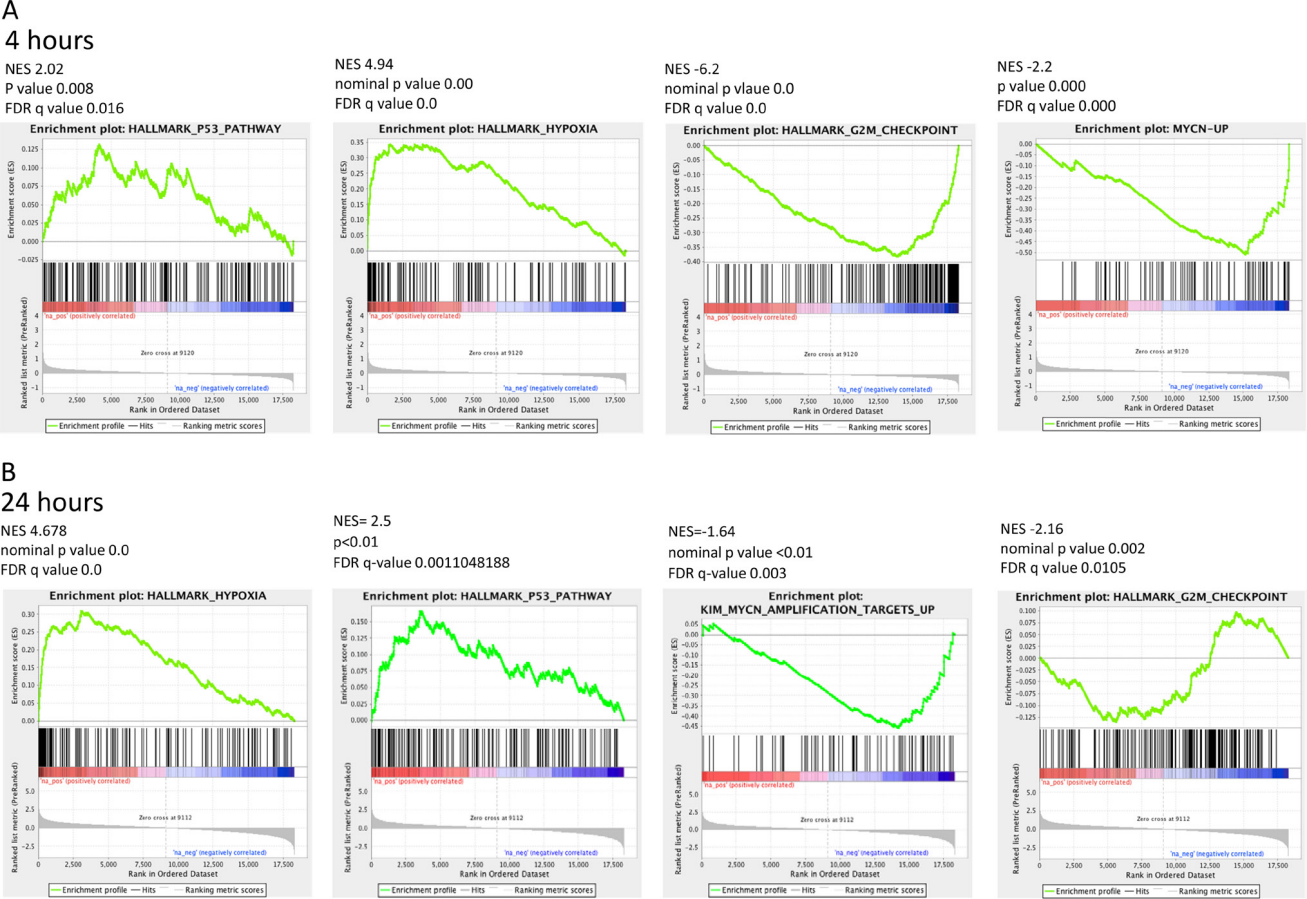
Significantly enriched GSEA signatures common and sustained at the 4- and 24- hour treatment time points GSEA for the Hallmark gene sets for transcriptional changes in NGP cells when treated with 3 µM HCI-2509 showed significant positive and negative enrichment for four gene sets that were common between the 4 and 24 hour time points. (**A**) GSEA plots at the 4 hour treatment time point. (**B**) GSEA plots at the 24 hour treatment time point.

**Figure 5:**
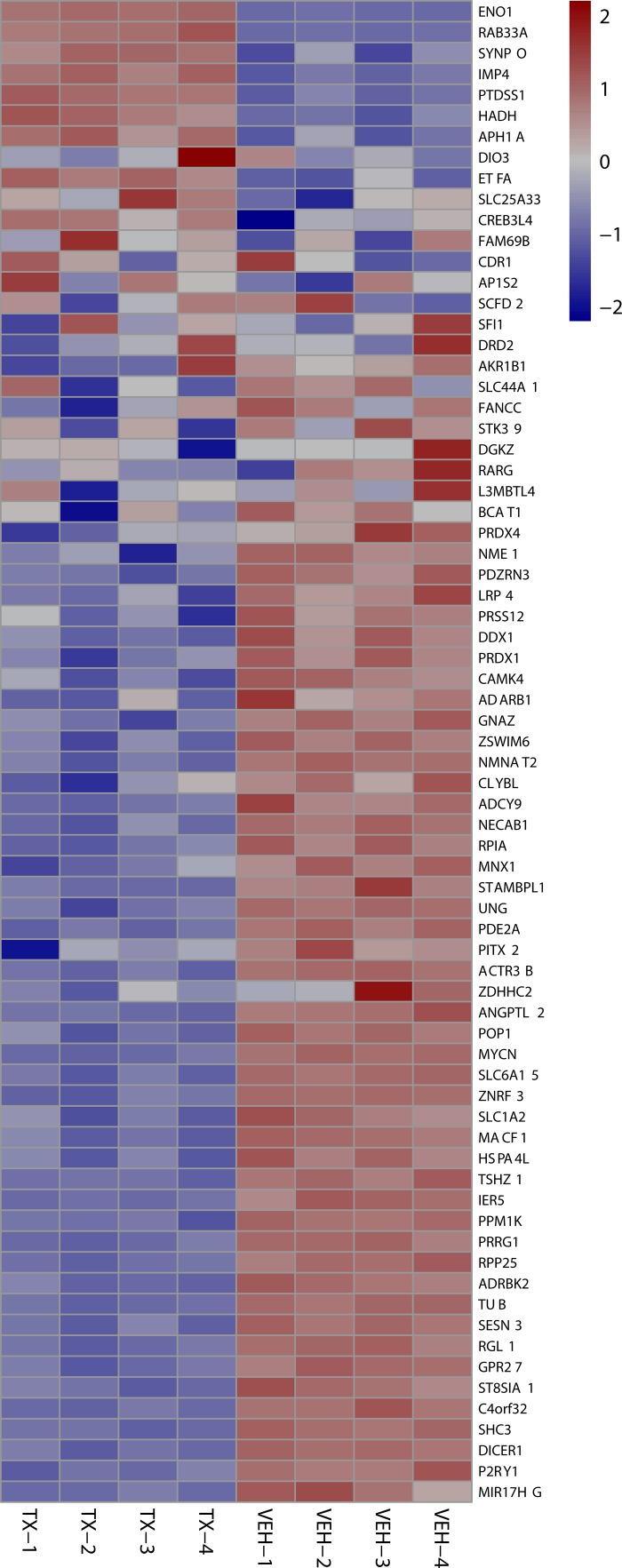
24 hour MYCN Up heat maps Heat map shows downregulation of MYCN amplification upregulated genes after 24 hours of 3 µM HCI-2509 treatment in NGP cells. Genes are sorted by fold change. (Veh-n = Vehicle treated cells replicate number, TX-n = HCI-2509 Treated cells replicate number).

The KIM MYCN amplification gene sets were identified among the Curated gene sets of MSigDB. The KIM MYCN amplification curated gene sets identify the gene sets upregulated and downregulated by MYCN amplification in small cell lung cancer cell lines. Gene Set Enrichment Analysis (GSEA) for this particular MYCN signature revealed that HCI-2509 leads to a significant downregulation of the genes that are upregulated by MYCN (FDR < 0.005) at both 4 and 24 hour treatment time points (Figures 4 and 5). Of note, there was no significant enrichment of the KIM MYCN amplification downregulated gene sets.

GSEA signatures for the Hallmark p53, Hypoxia, G2M checkpoint, and KIM MYCN Amplification Up gene sets are depicted in Figure 4A and 4B. All of these showed sustained enrichment through the two treatment time points in the same direction with an FDR *q* value that was significant and *p*-value that was significant. The Normalized Enrichment Scores (NES), *p*-values and FDR *q* values are presented with the GSEA signatures in Figure 4A and 4B.

**Figure 6:**
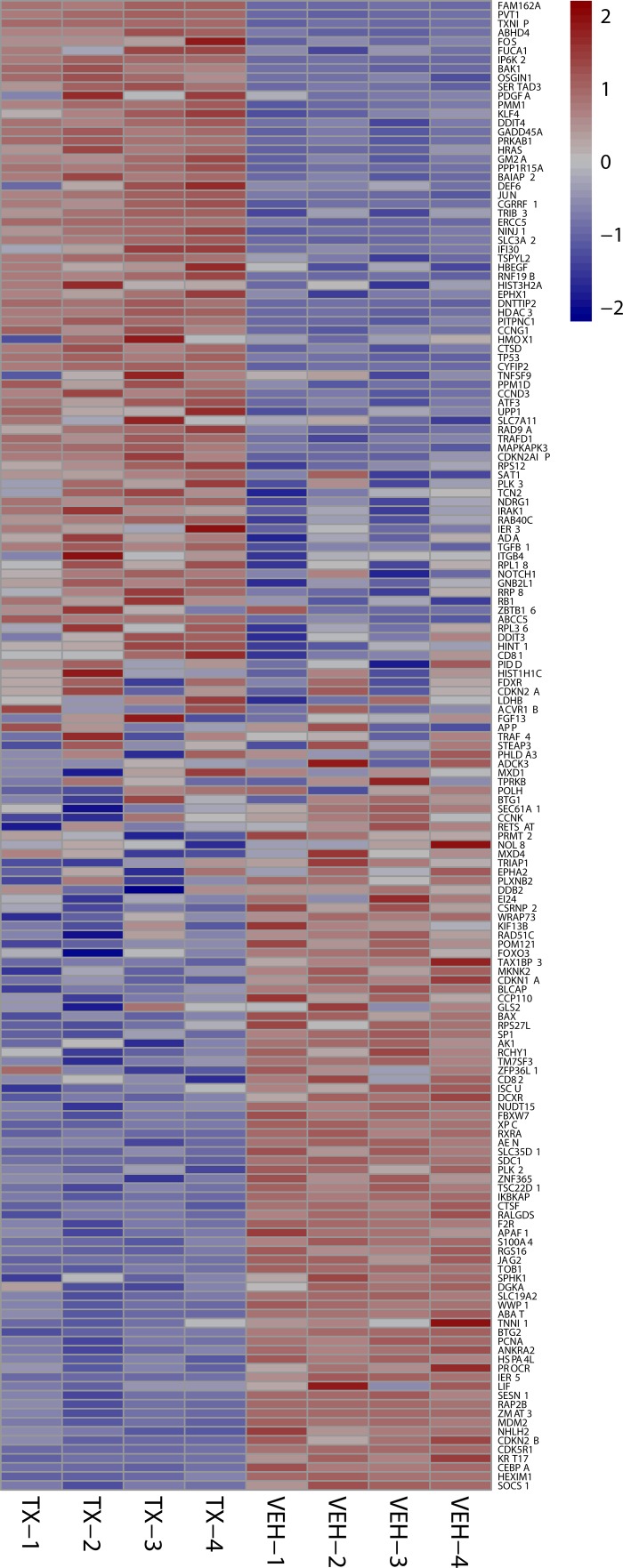
24 hour p53 signature heat maps Heat map shows the effect on P53 signature genes after 24 hours of HCI-2509 treatment in NGP cells. Genes are sorted by fold change. (Veh-n = Vehicle treated cells-replicate number, TX-n = HCI-2509 treated cells-replicate number).

Specifically the MYCN and p53 gene sets are particularly relevant in MYCN amplified neuroblastoma. About half of relapsed neuroblastoma have a defect in the P53 pathways. Majority of the these p53 pathways’ defects are upstream of *p53* gene and potentially targetable by agents that induce p53 [28, 29]. Gene expression profiling of NGP cells upon treatment with HCI2509 reveals a significant positive enrichment for the p53 gene signature and negative enrichment of the MYCN amplification up regulated genes at 4 and 24 hours (Figures 4 and 5). Heat maps to depict the gene expression change corresponding to the KIM MYCN Amplification Up gene set and the p53 gene set (genes involved in p53 pathways and networks) at 24 hours are shown in Figures 5 and 6, respectively.

### LSD1 inhibition with HCI-2509 affects gene expression along similar pathways in other neuroblastoma cell lines

**Figure 7:**
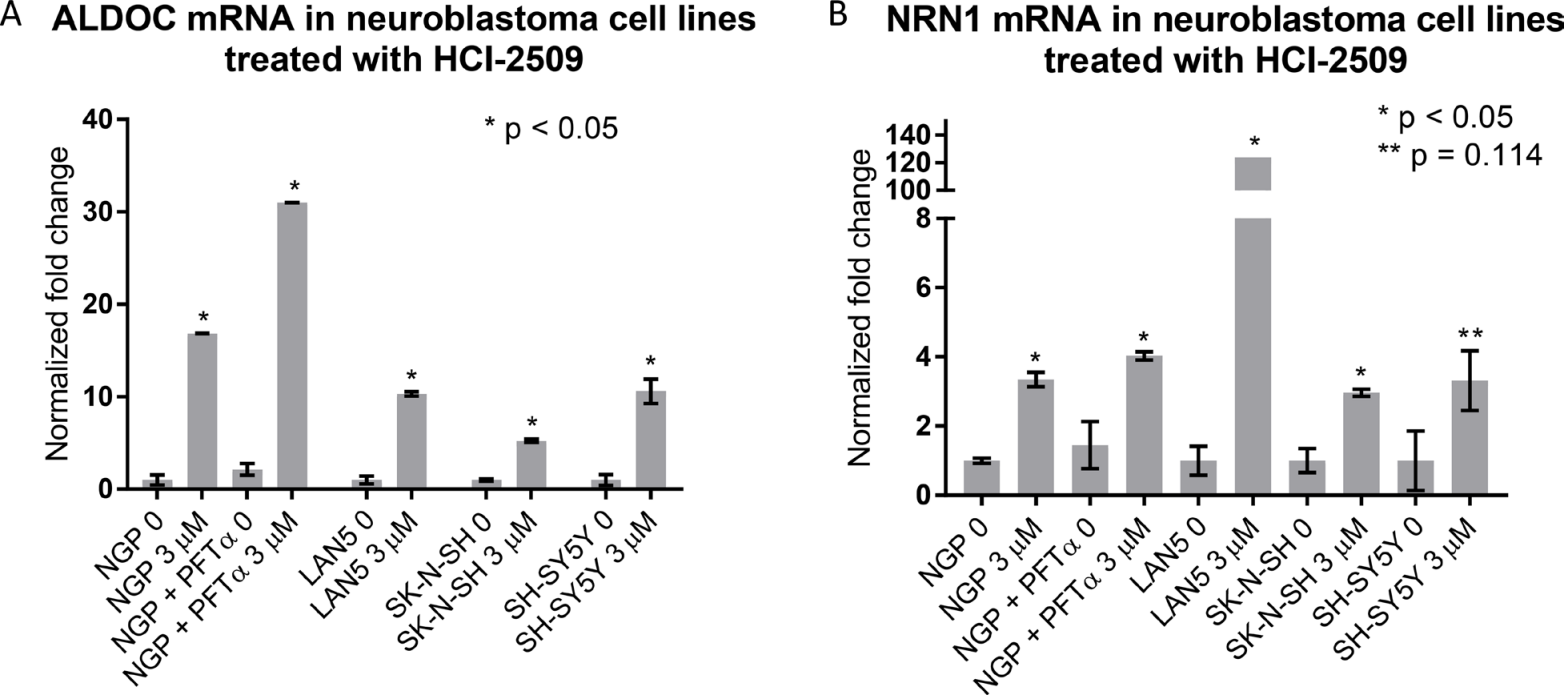
Gene expression changes in neuroblastoma cell lines treated with HCI-2509 assessed by quantitative reverse transcription-PCR (RTPCR) RNA was extracted from NGP, LAN5, SH-SY5Y and SK-N-SH cells treated with 0 or 3 µM HCI-2509 for 24 hours. Expression of (**A**) ALDOC and (**B**) NRN1 was assessed by RTPCR. NGP cells pretreated with 10 µM pifithrin-alpha (PFTα) were treated with HCI-2509 to assess p53 independent transcriptional effects of HCI-2509 on the same genes in NGP cells. Error bars represent biological replicates, significance tested by the unpaired *t*-test in GraphPad Prism. Bars are labeled as cell line+ treatment. 0 and 3 µM represent concentrations of HCI-2509. NGP cells pretreated with PFT-α are marked. An unpaired *t*-test for significance was performed between the difference in ALDOC expression with HCI-2509 in NGP cells pretreated with PFT-α and not pretreated with PFT-α had a *p* value of < 0.0001.

Increased expression of *ALDOC* and *NRN1* is noted in NGP cells treated with HCI-2509 (Figure 3). *ALDOC* expression represents activation of the glycolytic pathway (linked to hypoxia) and *NRN1* expression represents a state of post-mitotic differentiation of cells of neuronal origin, resulting in neurite growth. Quantitative reverse transcription and amplification with polymerase chain reaction (RT-PCR) for *ALDOC* and *NRN1* genes was performed on RNA extracted from all four cell lines (NGP, LAN5, SK-N-SH and SH-SY5Y) treated with HCI-2509 for 24 hours. A significant increase in expression of *ALDOC* was noted in all four cell lines treated with HCI-2509 (Figure 7A). *NRN1* expression was noted to be significantly increased in NGP, LAN5 and SK-N-SH cells and to a lesser degree in SH-SY5Y cells (Figure 7B). Specifically for the MCYN amplified NGP cell line, the stated gene expression changes with HCI-2509 were assessed after blockade of p53 effect with pifithrin-alpha (PFT-α) [30]. *ALDOC and NRN1,* gene expression changes with HCI-2509 remained consistent in the presence of PFT-α (Figure A and B). The increase in *ALDOC* expression is noted to be significantly (*p* < 0.0001) higher in the presence of PFT-α.

### HCI-2509 increases histone methyl marks on the fourth and ninth lysine residues of H3 and increases p53 levels in neuroblastoma cell lines

**Figure 8:**
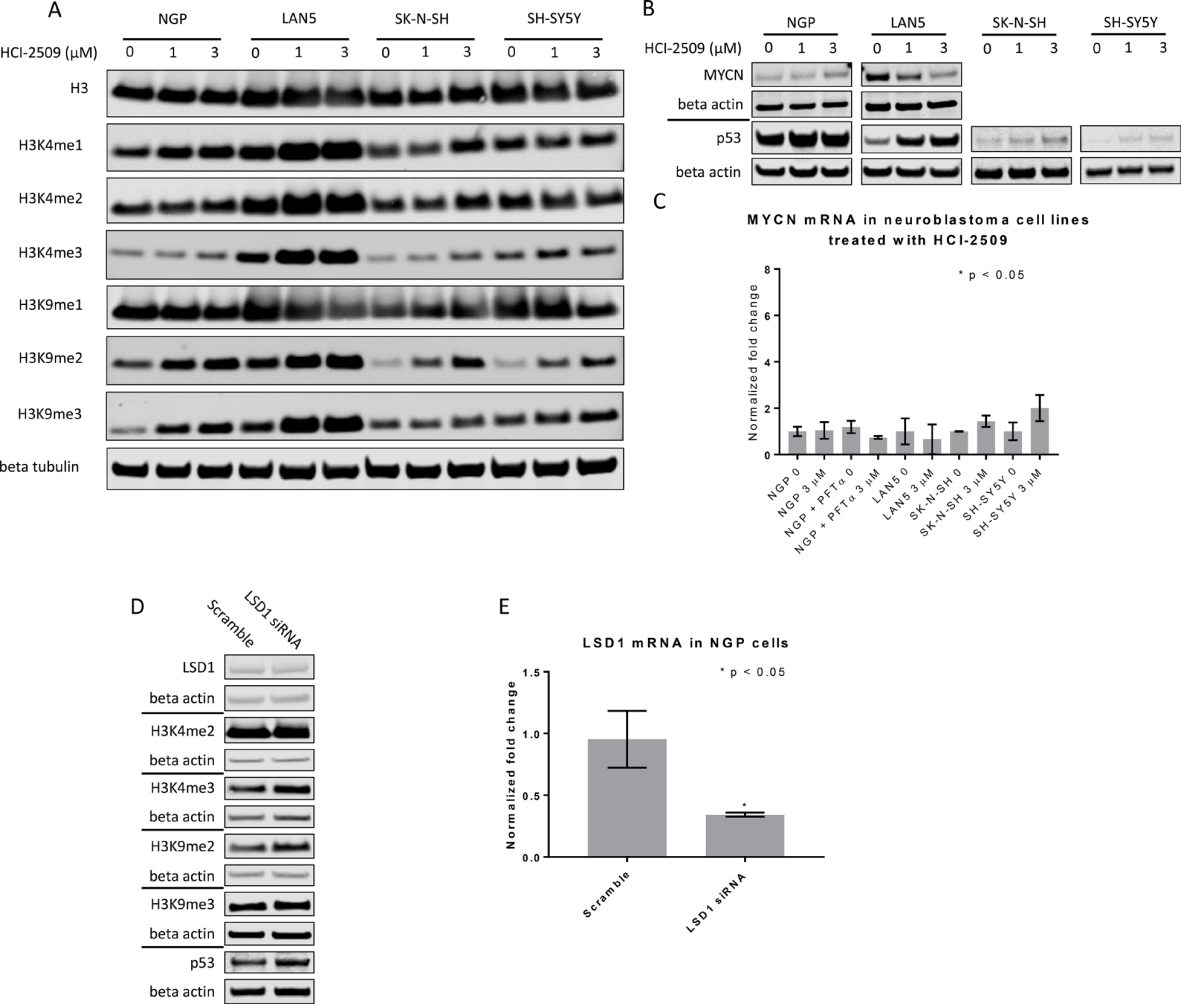
Western Blot and RTPCR to assess protein changes in neuroblastoma cell lines treated with HCI-2509 (**A**) Immunoblots to assess histone methylation in NGP, LAN5, and SH-SY5Y and SK-N-SH cells treated with 0, 1 and 3 µM of HCI-2509 for 48 hours. (**B**) P53 levels were assessed in all four cell lines treated with 0, 1 and 3 µM of HCI-2509 for 48 hours. MYCN levels were assessed in the MYCN-amplified NGP and LAN5 cells treated with 0, 1 and 3 µM of HCI-2509 for 48 hours. (**C**) RTPCR to assess MCYN expression in NGP, LAN5, SK-N-SH and SH-SY5Y cells upon treatment with HCI-2509 as compared to vehicle control (0). Additionally, NGP cells were pretreated with pifithrin-alpha (PFTα) and then were treated with 0 or 3 µM HCI-2509. Bars are labeled as cell line+ treatment. 0 and 3 µM represent concentrations of HCI-2509. NGP cells pretreated with PFT-α are marked. (**D**) Effect of LSD-1 siRNA as compared to scramble siRNA on p53, MYCN and histone methyl marks in NGP cells. (**E**) RTPCR demonstrating decreased transcription of LSD1 in NGP cells transfected with LSD1 siRNA as compared to scramble siRNA. Three biological replicates are represented with error bars and the relative inhibition of LSD1 expression was tested for significance by using the unpaired *t*-test (GraphPad Prism).

LSD1 is known to demethylate H3K4- and H3K9- di-and monomethyl marks [9, 11]. Consistent with these reports, a dose-dependent increase in H3K4me2 and H3K9me2 marks was observed in NGP, LAN5 and SK-N-SH cells that were treated with HCI-2509 (Figure 8A). In the case of SH-SY5Y cells, there is a dose dependent increase in H3K9me2 level, but not in the H3K4me2 level (Figure 8A). Downstream to this, H3K9me3 and H3K4me3 levels were noted to be increased upon treatment with HCI-2509 in all four cell lines (Figure 8A). A decrease in MYCN protein with HCI-2509 treatment was noted in LAN5 cells but not in NGP cells (Figure 8B). Additionally *MYCN* expression changes were assessed upon treatment with HCI-2509. No significant effect was noted in *MYCN* expression in NGP, LAN5, SK-N-SH and SH-SY5Ycells (Figure 8C).

A dose-dependent increase in p53 protein levels is noted in LAN5, SK-N-SH and SH-SY5Y cells upon treatment with HCI-2509 (Figure 8B). The p53 level in NGP cells is only slightly increased upon treatment with HCI-2509 (Figure 8B). LSD1 siRNA is noted to result in methylation of H3K9 and H3K4 residues and an increase in p53 levels as well (Figure 8D). There is no appreciable difference in MYCN levels with LSD1siRNA (Figure 8D).

### HCI-2509 results in G2M cell cycle arrest in NGP cells and early apoptosis in SK-N-SH cells

**Figure 9:**
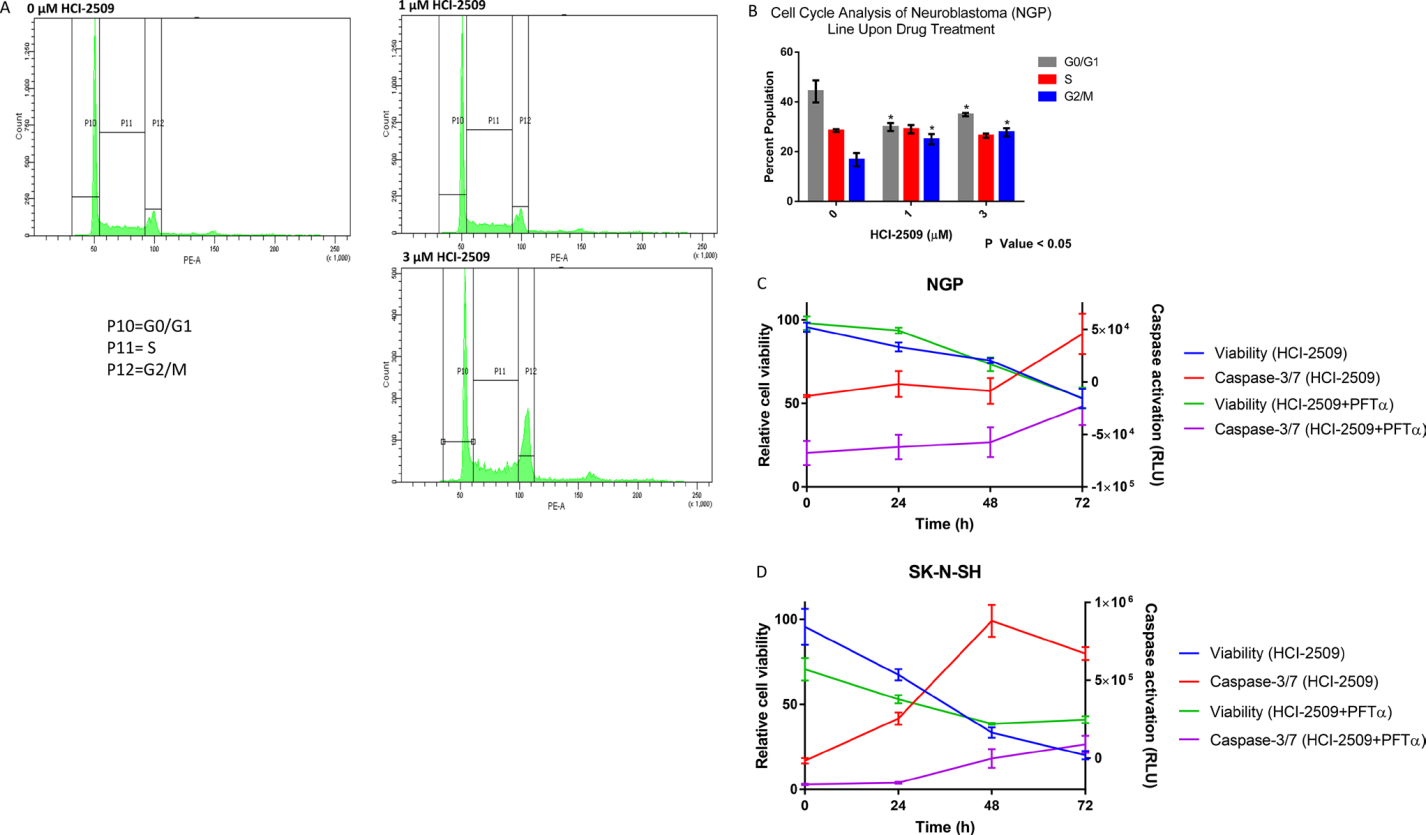
Cell cycle and viability changes and caspase activation with HCI-2509 (**A**) Cell cycle changes by flow cytometry in NGP cells were assessed at 24 hours after treatment with 0, 1 and 3 µM of HCI-2509. (**B**) GraphPad Prism was used to analyze the difference in the percent cell populations in the three phases of the cell cycle in untreated and treated samples. The change in the cell population distribution was replicated for a total of three times. An unpaired student *t*-test to compare the percent population with and without treatment showed the change in G0/G1 and G2/M populations to be statistically significant with treatment resulting in a decrease in the G0/G1 phase and increase in G2/M phase with *p*-value less than 0.05. (**C**) NGP cells treated with 3 µM HCI-2509 at 0, 24, 48 and 72 hours were assessed for viability and caspase activation by CellTiter-Glo and Caspase-Glo 3/7 assays, normalized to vehicle control and plotted as a function of time. Cells pretreated with PFTα were similarly treated and assessed. The error bars represent biological replicates.(**D**) SK-N-SH cells were similarly assessed for viability and caspase 3/7 activation with HCI-2509 at 0, 24 and 48 hours in the presence and absence of PFTα. The error bars represent biological replicates.

RNA seq and GSEA suggests multiple pathways affecting cell cycle progression as being significantly affected by HCI-2509 in NGP cells. To evaluate the effect of HCI-2509 on cell cycle progression, NGP cells were exposed to 1 µM and 3 µM of HCI-2509 for 24 hours. Flow cytometric analysis showed an increase in the G2M population (Figure 9A). An unpaired Student *t*-test to compare the percent population with and without treatment showed the change in G0/G1 and G2/M populations to be significant with a *p*-value <0.05. The difference in populations in the various phases of the cell cycle between the two treatment doses was not significant (Figure 9B).

To evaluate the degree of apoptotic cell death by caspase activation, a Caspase Glo 3/7 assay was performed in conjunction with cell viability assessment over a time course of treatment with HCI-2509.

NGP cells are noted to have a remarkable decrease in cell viability starting at 24 hours and progressively decreasing further up to 72 hours, but an indication of increased apoptosis by caspase 3/7 activation only at 72 hours (Figure 9C). On the other hand, the degree of early apoptosis at 24 hours, as indicated by increased levels of caspase 3/7, is remarkable in SK-N-SH cells (Figure D). To assess the role of p53 in the apoptotic cell death noted, we performed the same study in NGP and SK-N-SH cells pretreated with PFT-α and noted a reduction in the degree of caspase activation in both cell lines. The cell viability remained inhibited in NGP cells to the same extent and was less inhibited in SK-N-SH cells in the presence of PFT-α (Figure 9C and 9D).

### HCI-2509 inhibits colony formation of neuroblastoma cells

**Figure 10:**
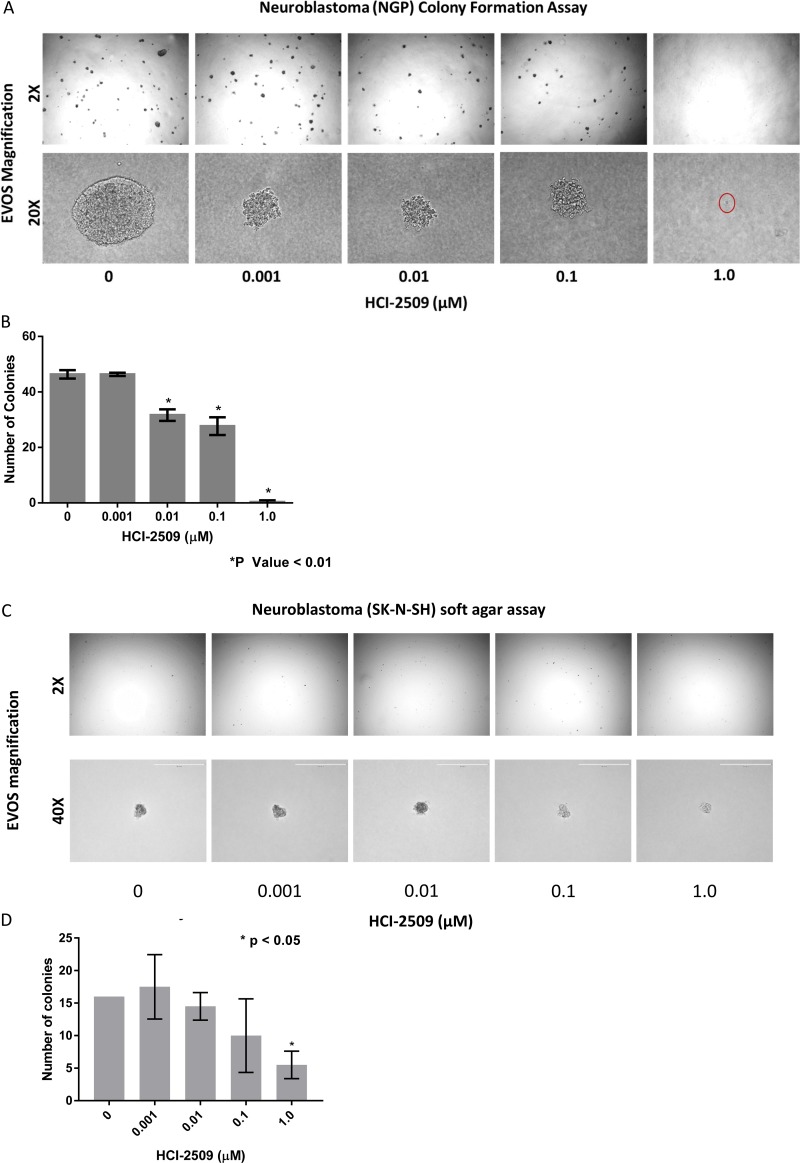
Colony formation assays (**A**) Colony Formation Assay: Microscopic appearance of NGP cell colonies treated with vehicle and increasing concentrations of HCI-2509 photographed at 2X and 20X magnification. (**B**) Bar graph representing the decrease on colony numbers on treatment as compared to control using Graphpad prizm. Error bars represent the standard error of mean (SEM) in duplicate assays. Statistical significance calculated by the unpaired student *t* test comparing the treated wells to vehicle wells. (**C**) Colony Formation Assay: Microscopic appearance of SK-N-SH cell colonies treated with vehicle and increasing concentrations of HCI-2509 photographed at 2X and 40X magnification. (**D**) Bar graph representing the decrease on colony numbers as compared to control using Graphpad prizm. Error bars represent the standard error of mean (SEM) in duplicate assays. Statistical significance calculated by the unpaired student *t* test comparing the treated wells to vehicle wells.

MYCN is a stem cell transcription factor and drives cell proliferation, survival and dedifferentiation in neuroblastoma cells. Given the significant negative enrichment for the MYCN upregulated gene sets in NGP cells treated with HCI-2509, we examined the anti-proliferative and stem cell inhibiting effects of HCI-2509 in colony formation assay. The growth of NGP cell colonies was suppressed in a dose-dependent manner, by size and number, upon treatment with HCI-2509. Moreover, a near complete ablation of colony formation was observed at 1 µM dose of HCI-2509 (Figure 10A, 10B).

On the other hand, non MYCN- amplified SK-N-SH cells were noted to have a significant reduction in colony formation only at 1 µM and not nearly as dramatic an effect on the colony size (Figure 10C, 10D).

It is to be noted that SK-N-SH colonies (Figure 10C and 10D), representing non-MYCN amplified neuroblastoma, were slower to grow and smaller in size than NGP cell colonies (Figure 10A and 10C).

## DISCUSSION

In this study, we have demonstrated that reversible, specific and potent LSD1 inhibition with HCI-2509 is growth inhibitory and cytotoxic to aggressive neuroblastoma cell lines. The IC50s are in high nanomole to low micromole range. LSD1 expression is independent of MYCN expression, and the 72 hour viability results are comparable in both MYCN-amplified and non-MYCN-amplified cell lines (Figure 1A, 1B), suggesting that cell death is mediated by mechanism(s) other than or more than abrogation of MYCN signaling. Gene expression profiling of NGP cells treated with HCI-2509 reveals a significant effect of LSD1 inhibition on multiple diverse gene sets. The effect on p53, hypoxia, G2M checkpoint and MYCN upregulated gene sets is the most sustained and explanatory of growth inhibition (Table 1, Figures 4, 5 and 6). Activation of gene expression belonging to hypoxia and differentiation pathways is noted in all four HCI-2509 treated cell lines studied by RTPCR. (Figure 7A and 7B).

Moreover, an increase in H3K4me2, H3K9me2, H3K4me3, H3K9me3 and p53 levels is noted in all four cell lines studied (Figure 8A, 8B). LSD1 is known to repress gene expression through demethylation of H3K4me1/2 in partnership with CoREST and NuRD. Specifically in the context of breast cancer and prostate cancer, LSD1 partners with nuclear hormone receptors to activate gene expression via demethylation of H3K9me1/2. In neuroblastoma cells, an isoform of LSD1 called LSD1+8a complexes with Supervillin protein (SVIL) and other co-factors. This complex harbors the specific binding capability to mediate demethylation of H3K9 me1/2. LSD1+8a is expressed in neuroblastoma and plays a major role in the process of neurite formation and differentiation during development [31]. Our results indicate that HCI-2509 likely targets the LSD1+8a isoform as well. LSD1 knockdown with siRNA corroborates the effect on H3K9 methylation as well (Figure 8D).

Long-term survival of children with treatment refractory neuroblastoma is approximately 8%. The low mutation rate distinguishes this disease entity from other solid tumors. Altered expressions of MYCN and ALK have been discussed as potential targets for novel therapeutic approaches [32]. MYCN gene amplification is a common event in untreated neuroblastomas, reported in as many as 38% of untreated patients, with a high degree of correlation with advanced disease stages and poor prognosis [33]. MYCN expression is exclusive to cells derived from the neural crest and determines migration and neuronal fate during embryonic development [34]. LSD1 is a binding partner of MYCN and influences the expression of tumor suppressor genes repressed by MYCN [19]. LSD1 inhibition has been shown to reduce MYCN-driven NDRG1 regulation, which affects epithelial-mesenchymal transition [20].

On specifically querying the effect of HCI-2509 on the transcriptional profile of NGP cells we demonstrate a significant downregulation of the MYCN upregulated gene set (Figures 4 and 5). There is no significant decrease in the MYCN protein and RNA levels in NGP cells with HCI-2509 treatment (Figure 8B and 8C). In LAN5 cells, MYCN protein reduction is noted in HCI-2509 treated cells, but MYCN gene expression does not change. It is possible that the effect of LSD1 inhibition on the MYCN signature may be related to the binding partnership between MYCN and LSD1 as a part of a transcriptionally influential complex [7, 19] driving the high-risk neuroblastoma signature.

As a stem cell maintenance factor, LSD1 plays a crucial role in embryogenesis. Along similar lines, it has been shown to maintain pluripotency and mediate EMT in the context of malignancy. Colony formation in an anchorage-independent manner in a soft agar medium represents the stem cell function of the cell that initiates the colony. The remarkable effect on colony formation with a significant reduction in the number of colonies of NGP cells (Figure 10A, 10B) shows a direct, on-target effect in poorly differentiated MYCN-amplified neuroblastoma of poor risk category. The dramatic effect on the size of the colonies also reflects an anti-proliferative effect of HCI2509 on the NGP cells. SK-N-SH cells in comparison to NGP cells did not exhibit as impressive colony formation. Moreover, inhibition of colony formation was also not as remarkable as was noted in NGP cells (Figure 10C, D). Our findings suggest a targeted benefit of LSD1 inhibition in MYCN driven neuroblastoma.

The arena of MYCN targeting has seen drugs targeting the DNA binding functions, transcription, synthetic interactions, oncogenic stabilization, and expression of MYCN. Epigenetic targeting of MYCN expression with BET bromodomain inhibitors and histone deacetylase inhibitors is effective in reducing MYCN levels in neuroblastoma [35]. Here we show that LSD1 inhibition with HCI-2509 has a potential role to play in downregulation of the MYCN upregulated genes of MYCN amplified neuroblastoma which translates to growth inhibitory effects.

LSD1 was initially discovered as a histone demethylase. LSD1 is known to modify histone proteins as well as non-histone proteins [36]. There has been a steady accumulation of evidence of the role of LSD1 in non-histone lysine demethylation such as in p53 and E2F1 induced cell death [37, 38]. LSD1 plays a role in the regulation of the tumor suppressor function of p53. Specifically, LSD1 demethylates K370me2 (the dimethylated 370th lysine residue of p53). K370me2 promotes association with coactivator 53BP1 (p53 –binding protein 1) through tandem Tudor domains in 53BP1. LSD1 represses p53 function by inhibiting this association. LSD1 inhibition allows p53 dimethylated at K370me2 to bind 53BP1 which facilitates p53 transcriptional activity [39]. The induction of p53 signature as seen in Figures 4A, 4B and 6 confirms a significant contribution of non-histone mediated effects of LSD1 inhibition by HCI-2509 in neuroblastoma. Along similar lines, LSD1 ablation and inhibition by tranylcypromine have previously been demonstrated to result in p53 mediated programmed cell death in the nerve cells of zebrafish embryos [40].

Neuroblastoma is typically p53 wildtype at diagnosis; however repression of p53 signaling mediated through MYCN and other mechanisms plays a major role in its pathogenesis. Treatment refractory neuroblastoma is characterized by inhibition of p53 activation mediated by oncogene-driven transcriptional networks. MDM2 plays a role in this inhibition of p53 leading to treatment resistance. Inhibition of MDM2 by Nutlin3a has been shown to activate p53 resulting in apoptosis [41, 42]. P53 has previously shown to be upregulated by MYCN [43]. MDM2, an inhibitor of p53, has also been shown to be upregulated by MYCN, leading to cell cycle progression and proliferation of neuroblastoma cells [44]. P53 mutations are very rare in neuroblastomas; however these do accumulate with progression through therapies [45]. P53 mediated cell death by LSD1 inhibition has clinical implications. Majority of P53 inactivation in relapsed tumors is upstream of the p53 gene and would likely be targetable by induction of the P53 pathway [28]. Nevertheless, around 15% percent of relapsed neuroblastomas have inactivating missense p53 mutations [28]. Does the lack of functional p53 take away the growth inhibitory or cytotoxic effects of LSD-1 inhibition with HCI-2509? To answer this question, we evaluated gene expression changes of the pathways affected by HCI-2509 in the presence of p53 inhibitor PFT-α. RTPCR does not show a significant change in the expression of HCI-2509 induced *ALDOC* and *NRN1* genes in the presence of PFT-α (Figure 7). We note that with use of p53 inhibitor PFT-α, neuroblastoma cells continue to have growth inhibition and cytotoxicity with HCI-2509 treatment with some loss in the degree of efficacy. Remarkably, the loss of cell viability with HCI-2509 is not affected in MYCN amplified NGP cells by removal of the p53 effect, but is blunted in the MCYN non-amplified SK-N-SH cells (Figure 9C, 9D). Our gene expression studies with HCI-2509 showed significant changes in pathways that influence cell cycle progression, including p53, MYCN and hypoxia. Cell cycle studies confirm inhibition of cell cycle progression with a net accumulation of cells in the G2/M phase of the cell cycle at 24 hours of treatment with HCI-2509 (Figure 9A, 9B). In p53 intact neoplastic cells, hypoxic stress results in increased p53 that leads to apoptosis [46]. Western Blot for p53 protein shows a dose-dependent increase in the p53 levels in LAN5, SK-N, SH and SH-SY5Y cells upon treatment with HCI-2509 (Figure 8B). The increase is p53 levels in NGP cells upon treatment with HCI-2509 is noted to be less prominent (Figure 8B). P53 induced cytotoxicity appears to play a bigger role in SK-N-SH cells than NGP cells as demonstrated in our caspase assays with PFT-α pretreated cells (Figure 9C and 9D). Based on our results, it appears that the growth of MYCN amplified NGP cells is affected by inhibition of the MYCN signature by HCI-2509, leading to decreased proliferation as seen prominently in the colony formation assay (Figure 10A and 10B). Hence, the role of p53 mediated caspase activation and cell death is not as prominent in NGP cells (Figure 9C). For SK-N-SH cells, in the absence of MYCN amplification, p53 mediated apoptotic cell death, as seen by caspase activation, appears to play a major role in early cytotoxicity from HCI-2509 (Figure 9D).

In conclusion, HCI-2509, a specific potent small molecule inhibitor of LSD1, inhibits the growth and causes cytotoxicity of poorly differentiated neuroblastoma cell lines mediated in part via p53. LSD1 inhibition can induce the p53 signature and may be a useful treatment strategy in poorly differentiated and relapsed neuroblastoma. A small subset of treatment-refractory neuroblastoma may not see this component of treatment benefit due to acquired inactivating p53 mutations. However, LSD-1 inhibition affects other pathways that contribute towards treatment effect. MYCN amplified high risk neuroblastoma, specifically, can be targeted by HCI-2509, as it affects both the p53 and the MYCN gene signatures.

## MATERIALS AND METHODS

### Methods

#### Cell culture and cell viability

SH-SY5Y, SK-N-SH, LAN5 and NGP neuroblastoma cell lines (donated by Rod Stewart lab) were cultured in DMEM supplemented with 10% FBS, 100 units/ml of penicillin, and 100ug/ml streptomycin. 5000 cells per well were seeded in 96 well plates in triplicate and after 24 hours were treated with increasing doses of HCI-2509. Cell viability was assessed with Cell Titer Glo (Promega) 72 hours after treatment. IC50s were calculated using GraphPad Prizm (GraphPad Software Inc., San Diego, USA). For further study of the effect of HCI-2509 on MYCN amplified cells we proceeded with the use of NGP cells. Cell line authentication of NGP cells was performed by STIR assay at ATCC.

### Transcriptomic profiling (RNA sequencing and analysis)

One million NGP cells per well were plated in 6 well plates and treated with vehicle (DMSO) or 3 µM HCI-2509 for 4 and 24 hours in quadruplicate. RNA was extracted using RNAeasy kit (Qiagen). Intact poly (A) RNA was purified from total RNA samples (100-500 ng) with oligo(dT) magnetic beads and sequencing libraries were prepared as described using the Illumina TruSeq RNA Library Preparation Kit v2 (RS-122-2001, RS-122-2002). Purified libraries were qualified on an Agilent Technologies 2200 TapeStation using a D1000 ScreenTape assay (cat# 5067-5582 and 5067-5583). The molarity of adapter-modified molecules was defined by quantitative PCR using the Kapa Biosystems Kapa Library Quant Kit (cat#KK4824). Individual libraries were normalized to 10 nM and equal volumes were pooled in preparation for Illumina sequence analysis. Sequencing libraries (18 pM) were denatured chemically and applied to an Illumina TruSeq v3 single read flowcell using an Illumina cBot. Using an Illumina TruSeq SR Cluster Kit v3-cBot-HS (GD-401-3001) hybridized molecules were clonally amplified and annealed to sequencing primers. Following transfer of the flowcell to an Illumina HiSeq instrument (HCS v2.0.12 and RTA v1.17.21.3), a 50 cycle single read sequence run was performed using TruSeq SBS v3 sequencing reagents (FC-401-3002).The transcriptome reference sequence was created by combining hg19 chromosomal sequences with splice junction sequences. The splice junction sequences from Ensembl (build 66) transcript annotations were generated with USeq (v8.6.8) MakeTranscriptome.

Using Novoalign (v2.08.01), reads were aligned to the transcriptome reference allowing up to 50 alignments for each read. USeq’s SamTranscriptomeParser application was used to select the best alignment location for each read and to convert the coordinates of reads aligning to splices back to genomic space. Approximately 80% of the reads aligned to the transcriptome and, of these, approximately 90% aligned to known genes (Ensembl annotations)

Differential gene expression was measured using USeq (v8.6.8) DefinedRegionDifferentialSeq [47]. The number of reads aligned to each gene were calculated. The counts were then used in DESeq2, which normalizes the signal and determines differential expression.

The DESeq2 function ‘vst’ was used to create variance stabilized, normalized counts. These counts were used in the DESeq2 function ‘plotPCA’ to create the Principal Component Analysis (PCA) using default settings. The Euclidean distances between each sample were generated by running the R function ‘dist’ on the counts. The resulting distances were displayed using the R package ‘pheatmap’.

Gene count data was transformed using the limma (v3.26.9) voom function [20–22]. The limma mroast function, set to 9,999 rotations, was used to test MYCN gene sets. The limma camera function was used to test all MSigDB Hallmark gene sets with the inter-gene correlation set to 0.01.

DESeq2 log2 fold change values were used to create GSEA rank files, which were then run in the GSEA pre-ranked tool to create enrichment plots for the MYCN and P53 gene sets.

Heat maps were generated using pheatmap (v1.0.8). Log2 FPKM values were mean-centered and divided by the standard deviation before plotting.

### Quantitative real-time RT-PCR

SH-SY5Y, SK-N-SH, LAN5 and NGP neuroblastoma cells were plated in 10 cm dishes at a density of one million per dish and left to adhere overnight. Once adherent, cells were treated with vehicle or 3 μM of HCI-2509 for 24 hours. To study the effect of P53 mediated apoptosis, a sub-population of NGP cells were cultured for 48 hour in the presence of 10 μM Pifithrin-α (Selleck) before being plated, at which point, Pifithrin-α was reapplied. RNA was extracted using RNeasy kit (Qiagen) and reverse transcribed to cDNA using High Capacity cDNA Reverse Transcription Kit (Applied Biosystems). cDNA was amplified, detected, and quantified with TaqMan Gene Expression Master Mix (Applied Biosystems) using ViiA7 Real-Time PCR System.

### Western blot

SH-SY5Y, SK-N-SH, LAN5 and NGP cells were plated in 10 cm dishes at a density of one million per dish and left to adhere overnight. Once adherent, cells were treated with vehicle, 1 μM and 3 μM of HCI-2509 for 48 hours. Protein was extracted from cell lysates and immunoblotted for methylated histone proteins, MYCN and p53.

### NGP LSD1 siRNA transfection

NGP cells were plated in 6 well plates at a density of 500, 000 cells per well and after 24 hours the growth media was switched to without antibiotics. Transfection with LSD1 siRNA and scramble siRNA at a concentration of 50 nM was performed using lipofectamine in optiMEM. After incubation for 48 hours, cells were harvested. RNA was extracted to assess LSD-1 expression and protein was extracted for immunoblotting.

### Cell cycle study

NGP cells were treated with vehicle, or 1 or 3 µM of HCI-2509 for 24 hours. The cells were collected, fixed in ethanol and stained with Propidium Iodide. Cell Cycle analysis was performed using FACS Canto flow cytometer. The experiment was replicated two more times, and the differences in cell population fractions were plotted using GraphPad Prism. The percentage of cell population in the G1/G0, S and G2/M phases of cell cycle were compared in the vehicle treated and HCI-2509 1 and 3 µM treated samples. The difference in the distribution of cells in the different phases of the cell cycle in the three different conditions was tested for significance by performing a Student unpaired *t*-test using GraphPad Prism.

### *In vitro* apoptosis and viability assay

20,000 cells per well were seeded in 96 well plates in triplicate and after 24 hours were treated with vehicle or 3 μM HCI-2509 for 72, 48, 24, and 0 hours(for NGP cells) or 48, 24 and 0 hours(for SK-N-SH cells). To study the effect of P53 mediated apoptosis, a sub-population of NGP and SK-N-SH cells were cultured for 48 hours in the presence of 10 μM Pifithrin-α before being plated, at which point, Pifithrin-α was reapplied. Caspase 3/7 activity was assayed using Caspase-Glo 3/7 (Promega) and cell viability was determined with CellTiter-Glo (Promega).

### Colony formation assay

NGP and SK-N-SH cells treated with vehicle and varying concentrations of HCI-2509 (1nM to 1000nM) were seeded in 0.35% agarose (Sea Plaque) with a solidified cell-free under layer of 0.7 % agarose in 6 well plates. The cells were seeded at a density of 5000 cells per well and followed for colony growth over 14 to 21 days without additional drug treatment. Colonies were photographed and counted.

### Materials

#### Primers

The following TaqMan gene expression assay primers were used:

*ALDOC* (Hs00902799_g1), *NRN1* (Hs00213192_m1), *MYCN* (Hs00232074_m1), *B2M* (Hs0000187842_m1). *LSD1* Primer; ThermoFisher KDM1A Assay ID: Hs01002741_m1 Cat#: 4453320.

### Antibodies

Immunodetection was performed with the following antibodies:

### Cell Signaling

Histone H3 (D1H2) XP(TM) Rabbit mAb 4499S; Mono-Methyl-Histone H3 (K4) (D1A9) XP(R) Rabbit mAb 5326S; Di-Methyl-Histone H3 (K4) (C64G9) Rabbit mAb 9725S; Tri-Methyl-Histone H3 (K4) (C42D8) Rabbit mAb 9751S; Mono-Methyl-Histone H3 (K9) (D1P5R) Rabbit mAb 14186S; Di-Methyl-Histone H3 (K9) (D85B4) XP(R) Rabbit mAb 4658S; Tri-Methyl-Histone H3 (Lys9) (D4W1U) Rabbit mAb 13969S; beta-Actin (13E5) Rabbit mAb 4970S; beta-Tubulin (D3U1W) Mouse mAb 86298S; P53 Rabbit Ab #9282S; LSD1 (C69G12) Rabbit mAb #2184S.

### Abcam

Mouse mAb to n-Myc [NCM II 100] ab16898.

### siRNA

LSD1 SiRNA: Ambion Cat^#^: 4390827.

### Negative control

Silencer – Negative Control siRNA ^#^1 – Ambion AM4611 – 50 uM concentration.

## Abbreviations

LSD1: Lysine Specific Demethylase 1
H3K4me2: dimethylated fourth lysine on Histone 3
H3K4me1: monomethylated fourth lysine on Histone 3
H3K9me2: dimethylated ninth lysine on Histone 3
H3K9me1: monomethylated ninth lysine on Histone 3

## References

[1] National Cancer Institute Neuroblastoma Treatment (PDQ^®^)–Health Professional Version. General information about neuroblastoma.

[2] Maris JM (2010). Recent advances in neuroblastoma. N Engl J Med.

[3] National Cancer Institute https://seer.cancer.gov/csr/1975_2014.

[4] American Cancer Society Survival rates for neuroblastoma based on risk groups. https://www.cancer.org/cancer/neuroblastoma/detection-diagnosis-staging/survival-rates.html.

[5] Valentijn LJ, Koster J, Haneveld F, Aissa RA, van Sluis P, Broekmans ME, Molenaar JJ, van Nes J, Versteeg R (2012). Functional MYCN signature predicts outcome of neuroblastoma irrespective of MYCN amplification. Proc Natl Acad Sci USA.

[6] Kobayashi K, Jakt LM, Nishikawa SI (2013). Epigenetic regulation of the neuroblastoma genes, Arid3b and Mycn. Oncogene.

[7] Huang M, Weiss WA (2013). Neuroblastoma and MYCN. Cold Spring Harb Perspect Med.

[8] Shi Y, Lan F, Matson C, Mulligan P, Whetstine JR, Cole PA, Casero RA, Shi Y (2004). Histone demethylation mediated by the nuclear amine oxidase homolog LSD1. Cell.

[9] Ballas N, Battaglioli E, Atouf F, Andres ME, Chenoweth J, Anderson ME, Burger C, Moniwa M, Davie JR, Bowers WJ, Federoff HJ, Rose DW, Rosenfeld MG (2001). Regulation of neuronal traits by a novel transcriptional complex. Neuron.

[10] Wang Y, Zhang H, Chen Y, Sun Y, Yang F, Yu W, Liang J, Sun L, Yang X, Shi L, Li R, Li Y, Zhang Y (2009). LSD1 is a subunit of the NuRD complex and targets the metastasis programs in breast cancer. Cell.

[11] Metzger E, Wissmann M, Yin N, Müller JM, Schneider R, Peters AH, Günther T, Buettner R, Schüle R (2005). LSD1 demethylates repressive histone marks to promote androgen-receptor-dependent transcription. Nature.

[12] Adamo A, Sesé B, Boue S, Castaño J, Paramonov I, Barrero MJ, Izpisua Belmonte JC (2011). LSD1 regulates the balance between self-renewal and differentiation in human embryonic stem cells. Nat Cell Biol.

[13] Zheng YC, Ma J, Wang Z, Li J, Jiang B, Zhou W, Shi X, Wang X, Zhao W, Liu HM (2015). A systematic review of histone lysine-specific demethylase 1 and its inhibitors. Med Res Rev.

[14] Amente S, Lania L, Majello B (2013). The histone LSD1 demethylase in stemness and cancer transcription programs. Biochim Biophys Acta.

[15] Schulte JH, Lim S, Schramm A, Friedrichs N, Koster J, Versteeg R, Ora I, Pajtler K, Klein-Hitpass L, Kuhfittig-Kulle S, Metzger E, Schüle R, Eggert A (2009). Lysine-specific demethylase 1 is strongly expressed in poorly differentiated neuroblastoma: implications for therapy. Cancer Res.

[16] Xu G, Xiao Y, Hu J, Xing L, Zhao O, Wu Y (2013). The combined effect of retinoic acid and LSD1 siRNA inhibition on cell death in the human neuroblastoma cell line SH-SY5Y. Cell Physiol Biochem.

[17] Althoff K, Beckers A, Odersky A, Mestdagh P, Köster J, Bray IM, Bryan K, Vandesompele J, Speleman F, Stallings RL, Schramm A, Eggert A, Sprüssel A, Schulte JH (2013). MiR-137 functions as a tumor suppressor in neuroblastoma by downregulating KDM1A. Int J Cancer.

[18] Han X, Gui B, Xiong C, Zhao L, Liang J, Sun L, Yang X, Yu W, Si W, Yan R, Yi X, Zhang D, Li W (2014). Destabilizing LSD1 by Jade-2 promotes neurogenesis: an antibraking system in neural development. Mol Cell.

[19] Amente S, Milazzo G, Sorrentino MC, Ambrosio S, Di Palo G, Lania L, Perini G, Majello B (2015). Lysine-specific demethylase (LSD1/KDM1A) and MYCN cooperatively repress tumor suppressor genes in neuroblastoma. Oncotarget.

[20] Ambrosio S, Amente S, Saccà CD, Capasso M, Calogero RA, Lania L, Majello B (2017). LSD1 mediates MYCN control of epithelial-mesenchymal transition through silencing of metastatic suppressor NDRG1 gene. Oncotarget.

[21] Sorna V, Theisen ER, Stephens B, Warner SL, Bearss DJ, Vankayalapati H, Sharma S (2013). High-throughput virtual screening identifies novel N′-(1-phenylethylidene)-benzohydrazides as potent, specific, and reversible LSD1 inhibitors. J Med Chem.

[22] Sankar S, Theisen ER, Bearss J, Mulvihill T, Hoffman LM, Sorna V, Beckerle MC, Sharma S, Lessnick SL (2014). Reversible LSD1 inhibition interferes with global EWS/ETS transcriptional activity and impedes Ewing sarcoma tumor growth. Clin Cancer Res.

[23] Theisen ER, Gajiwala S, Bearss J, Sorna V, Sharma S, Janat-Amsbury M (2014). Reversible inhibition of lysine specific demethylase 1 is a novel anti-tumor strategy for poorly differentiated endometrial carcinoma. BMC Cancer.

[24] Gupta S, Weston A, Bearrs J, Thode T, Neiss A, Soldi R, Sharma S (2016). Reversible lysine-specific demethylase 1 antagonist HCI-2509 inhibits growth and decreases c-MYC in castration- and docetaxel-resistant prostate cancer cells. Prostate Cancer Prostatic Dis.

[25] Abemayor E, Sidell N (1989). Human neuroblastoma cell lines as models for the *in vitro* study of neoplastic and neuronal cell differentiation. Environ Health Perspect.

[26] Puissant A, Frumm SM, Alexe G, Bassil CF, Qi J, Chanthery YH, Nekritz EA, Zeid R, Gustafson WC, Greninger P, Garnett MJ, McDermott U, Benes CH (2013). Targeting MYCN in neuroblastoma by BET bromodomain inhibition. Cancer Discov.

[27] Gamble LD, Kees UR, Tweddle DA, Lunec J (2012). MYCN sensitizes neuroblastoma to the MDM2-p53 antagonists Nutlin-3 and MI-63. Oncogene.

[28] Carr-Wilkinson J, O’Toole K, Wood KM, Challen CC, Baker AG, Board JR, Evans L, Cole M, Cheung NK, Boos J, Köhler G, Leuschner I, Pearson AD (2010). High frequency of p53/MDM2/p14ARF pathway abnormalities in relapsed neuroblastoma. Clin Cancer Res.

[29] Carr J, Bell E, Pearson AD, Kees UR, Beris H, Lunec J, Tweddle DA (2006). Increased frequency of aberrations in the p53/MDM2/p14(ARF) pathway in neuroblastoma cell lines established at relapse. Cancer Res.

[30] Rocha S, Campbell KJ, Roche KC, Perkins ND (2003). The p53-inhibitor pifithrin-α inhibits firefly luciferase activity *in vivo* and *in vitro*. BMC Mol Biol.

[31] Laurent B, Ruitu L, Murn J, Hempel K, Ferrao R, Xiang Y, Liu S, Garcia Benjamin A, Wu H, Wu F, Steen H, Shi Y A specific LSD1/KDM1A isoform regulates neuronal differentiation through H3K9 demethylation. Molecular Cell.

[32] Barone G, Anderson J, Pearson AD, Petrie K, Chesler L (2013). New strategies in neuroblastoma: therapeutic targeting of MYCN, ALK. Clin Cancer Res.

[33] Brodeur GM, Seeger RC, Schwab M, Varmus HE, Bishop JM (1984). Amplification of N-myc in untreated human neuroblastomas correlates with advanced disease stage. Science.

[34] Wakamatsu Y, Watanabe Y, Nakamura H, Kondoh H (1997). Regulation of the neural crest cell fate by N-myc: promotion of ventral migration and neuronal differentiation. Development.

[35] Shahbazi J, Liu PY, Atmadibrata B, Bradner JE, Marshall GM, Lock RB, Liu T (2016). The bromodomain inhibitor JQ1 and the histone deacetylase inhibitor panobinostat synergistically reduce N-Myc expression and induce anticancer effects. Clin Cancer Res.

[36] Kooistra SM, Helin K (2012). Molecular mechanisms and potential functions of histone demethylases. Nat Rev Mol Cell Biol.

[37] Huang J, Sengupta R, Espejo AB, Lee MG, Dorsey JA, Richter M, Opravil S, Shiekhattar R, Bedford MT, Jenuwein T, Berger SL (2007). p53 is regulated by the lysine demethylase LSD1. Nature.

[38] Kontaki H, Talianidis I (2010). Lysine methylation regulates E2F1-induced cell death. Mol Cell.

[39] Scoumanne A, Chen X (2008). Protein methylation: a new regulator of the p53 tumor suppressor. Histol Histopathol.

[40] Jie Z, Li T, Jia-Yun H, Qiu J, Ping-Yao Z, Houyan S (2009). -2-phenylcyclopropylamine induces nerve cells apoptosis in zebrafish mediated by depression of LSD1 activity. Brain Res Bull.

[41] Barbieri E, De Preter K, Capasso M, Johansson P, Man TK, Chen Z, Stowers P, Tonini GP, Speleman F, Shohet JM (2013). p53 drug response signature identifies prognostic genes in high-risk neuroblastoma. PLoS One.

[42] Van Maerken T, Ferdinande L, Taildeman J, Lambertz I, Yigit N, Vercruysse L, Rihani A, Michaelis M, Cinatl J, Cuvelier CA, Marine JC, De Paepe A, Bracke M (2009). Antitumor activity of the selective MDM2 antagonist nutlin-3 against chemoresistant neuroblastoma with wild-type p53. J Natl Cancer Inst.

[43] Chen L, Iraci N, Gherardi S, Gamble LD, Wood KM, Perini G, Lunec J, Tweddle DA (2010). p53 is a direct transcriptional target of MYCN in neuroblastoma. Cancer Res.

[44] Slack A, Chen Z, Tonelli R, Pule M, Hunt L, Pession A, Shohet JM (2005). The p53 regulatory gene MDM2 is a direct transcriptional target of MYCN in neuroblastoma. Proc Natl Acad Sci USA.

[45] Imamura J, Bartram CR, Berthold F, Harms D, Nakamura H, Koeffler HP (1993). Mutation of the p53 gene in neuroblastoma and its relationship with N-myc amplification. Cancer Res.

[46] Leszczynska KB, Foskolou IP, Abraham AG, Anbalagan S, Tellier C, Haider S, Span PN, O’Neill EE, Buffa FM, Hammond EM (2015). Hypoxia-induced p53 modulates both apoptosis and radiosensitivity via AKT. J Clin Invest.

[47] Love MI, Huber W, Anders S (2014). Moderated estimation of fold change and dispersion for RNA-seq data with DESeq2. Genome Biol.

